# On-Field Perceptual-Cognitive Training Improves Peripheral Reaction in Soccer: A Controlled Trial

**DOI:** 10.3389/fpsyg.2020.01948

**Published:** 2020-08-07

**Authors:** Nils Schumacher, Rüdiger Reer, Klaus-Michael Braumann

**Affiliations:** Faculty of Psychology and Human Movement, Institute of Human Movement Science, University of Hamburg, Hamburg, Germany

**Keywords:** cognition, expert performance, perceptual-cognitive training, visual perception, expert-performance approach, peripheral reaction, lateralization, cognitive component skill approach

## Abstract

Abilities such as peripheral reaction are of special importance in soccer. Whether these abilities can be improved by sport-specific on-field interventions remains unclear. The aim of the present controlled trial was to investigate the effect of a soccer-specific perceptual-cognitive on-field training on peripheral reaction of highly talented soccer players aged 12–13 years. *N* = 38 male elite athletes from young talent centers were allocated to an intervention (*n* = 19) and a control group (CG) (*n* = 19). Computer-based peripheral perception tests were conducted before and after intervention. Combining a sport-specific and a juggling task, the intervention was performed once a week (8 weeks, 20 min per week) in addition to team training. The CG exclusively underwent usual team training. Analyses show significant differences between the two groups for peripheral reaction time (PRT), with significant improvements for the intervention group and none for the CG. Furthermore, results indicate that improvements in peripheral reaction might be due to changes in the reaction time of right-footed players. Future studies should be conducted to clarify the effect of sport-specific on-field training approaches on PRT. These analyses should consider the influence of lateralization on effectivity of perceptual-cognitive on-field training approaches.

## Introduction

The importance of perceptual-cognitive abilities in sport games has been investigated in various studies (Zwierko, 2007; Ryu et al., 2013; Poltavski and Biberdorf, 2015). In many sports, especially in team ball games such as soccer, abilities such as peripheral perception seem to be of special interest (Ando et al., 2001; Vater et al., 2019a). The players must pay attention not only to the movement of the ball, but also to opponents and teammates in their visual field. Nevertheless, the trainability of abilities such as peripheral perception with sport-specific on-field methods remains to be determined.

The visual field is composed of both central/foveal vision and peripheral vision (Magill, 2007b). Central/foveal vision only covers approximately the central 1.7° eccentricity of the visual field (Rosenholtz, 2016). Peripheral vision detects information in the visual field outside these limits, thus about 99.9% of the visual field (Magill, 2007b; Rosenholtz, 2016). On the basis of peripheral as well as central visual information, the soccer players are often required to decide and react as fast as possible. The interval of time between the onset of such a signal in the visual field and the initiation of a response is defined as reaction time (Magill, 2007a).

The reaction to visual stimuli from the periphery might be a key component of performance in soccer. Studies investigated differences between experts and novices concerning peripheral visual reaction time in ball sports (Ando et al., 2001; Zwierko, 2007). Ando et al. (2001) measured the nervous systems processing time, defined as premotor time, and muscle contraction time, defined as motor time, using electromyogram during peripheral visual reaction tasks in soccer players. They showed that experts had shorter premotor times during central and peripheral visual reaction tasks than novices (Ando et al., 2001). Using the computer-based Vienna Test System, Zwierko (2007) reported that handball players had significantly shorter response time to stimuli appearing in the peripheral field of vision (FOV) compared to non-athletes. These results suggest that experts in ball sports such as soccer have shorter premotor times during central and peripheral visual reaction tasks than non-athletes. In addition, these findings indicate a relation between expertise in soccer and the peripheral response time. Thus, for talent promotion, analyses about the efficacy of perceptual-cognitive training approaches on peripheral reaction might be of special interest and could deliver several approaches for practical applications. Investigations on the effect of sport-specific on-field training on general perceptual-cognitive abilities may also be of interest in the context of two broad theories in expert performance research, namely, the *expert-performance approach* and the *cognitive component skill approach*. The cognitive component skill approach examines the relationship between sports expertise and performance of non–sport-specific, general cognition (Nougier et al., 1991). The expert-performance approach analyzes the experts under a sport-specific, ecologically valid context (Gilbert et al., 2016). Analyses on the effect of a sport-specific on-field training on general perceptual abilities could contribute to a better understanding of sporting expertise and may allow a discussion on the expert-performance approach in the context of the cognitive component skill approach.

Research has shown that perceptual-cognitive skills can be trained in sports (Savelsbergh et al., 2010; Schwab and Memmert, 2012; Romeas et al., 2016; Larkin et al., 2018; Brenton et al., 2019). For example, Romeas et al. (2016) assessed the transferability of a computer-based perceptual-cognitive three-dimensional training from a laboratory setting to a soccer field. The experimental group conducted two sessions per week (5 weeks, 8 min per session). They demonstrated that decision-making accuracy in passing, but not in dribbling and shooting, between presessions and postsessions was superior for the three-dimensional trained group compared to control groups (CGs). Nevertheless, the small sample size, the subjective judgments of response accuracy, and a non-validated transfer test must be mentioned. Savelsbergh et al. (2010) conclude that after a computer-based training intervention the visual search behavior of the perceptual training group improved significantly. Schwab and Memmert (2012) investigated whether a sports vision training program improved the visual performance of youth male field hockey players after an intervention of 6 weeks compared to a CG with no specific sports vision training. A choice reaction time task, the functional field of view task, and the multiple-objects tracking task were examined before and after the intervention and again 6 weeks after the second test. They discuss significant differences between the intervention and CG for the choice reaction time task at the D2 board and the functional field of view task, indicating significant improvements for the intervention group (IG).

Nevertheless, there remains a need for studies investigating the effect of a sport-specific on-field training. This seems particularly relevant as it was also suggested that practical training approaches should consider ecological validity, transferability, and *perception action-coupling* (Broadbent et al., 2015a, b; Hadlow et al., 2018; Renshaw et al., 2019). To our knowledge no studies investigating the effect of a sport-specific on-field perceptual-cognitive training on peripheral perception and especially peripheral reaction exist to date. In order to take this into account, in the present study, we combined a sport specific on-field with a juggling task.

Earlier studies analyzed the role of juggling for gaze behavior and peripheral vision (Beek and Lewbel, 1995; Huys and Beek, 2002; Beek et al., 2003). It was shown that juggling expertise was associated with an overall reduction in the degree to which the balls were visually tracked. They stated that visual behavior and the use of peripheral vision vary depending on expertise and that probably intermediate jugglers relied more on peripheral vision than the inexperienced or expert jugglers. Moreover, investigations of the primary motor cortex on hemodynamic responses during juggling tasks indicated that a higher level of expertise might be associated with lower hemodynamic responses (Carius et al., 2016). These results suggest that juggling expertise might have an influence on peripheral vision during complex motor tasks and potentially on underlying mechanisms.

The purpose of the present study was to investigate the effect of a soccer-specific perceptual-cognitive on-field training combining a sport-specific and a juggling task on the peripheral reaction abilities of highly talented soccer players. Male athletes (*N* = 38) were recruited from young talent centers of a German first and a second league soccer club. We hypothesized that an 8-week perceptual-cognitive on-field training intervention improves the peripheral reaction time (PRT) of elite male soccer players.

## Materials and Methods

### Participants

Based on previous studies on perceptual-cognitive training (Schwab and Memmert, 2012), we expected a medium effect size (*f*^2^ = 0.25; α = 0.05, and β = 0.80). An *a priori* sample size calculation for the 2 × 2 factorial analysis of variance (ANOVA) for repeated-measures (within–between interaction) using G^∗^Power version 3.1.9.4 determined a sample size of *N* = 34. Under consideration, of assumed dropout of 10%, two teams of highly talented male soccer players (*N* = 38) were recruited from young talent centers of German professional soccer clubs (first and a second league). Both teams played in the same league at the highest level of national competition for their age group (under 14 (U 14)). To distinguish between high−performance and amateur athletes, Scharfen and Memmert (2019) suggest the “elite” rather than the “expert” definition. Therefore, we classified the athletes based on their current competition level for their age group. Inclusion criteria were male soccer players between 12 and 13 years with a minimum of 5 years of experience in soccer, playing at the highest level of national competition in their age group (U 14). All subjects reported normal visual acuity either unaided or while wearing their own corrective lenses and had no prior experience with computer-based peripheral vision measurements. The players presented no juggling skills in both groups prior to the intervention and stated that they had a similar daily routine at *t*_0_ and *t*_1_.

### Ethics Statement

Ethical approval for the present study was obtained from the local ethics committee of the Faculty of Psychology and Human Movement Science, University of Hamburg (AZ 2017_106). Data collection took place anonymously, and all measurements were non-invasive. Prior to the study, all participants and/or legal guardians gave their written informed consent. Only participants following these rules were included in the study. The study followed the principles of the Helsinki Declaration.

### Design

**FIGURE 1:**
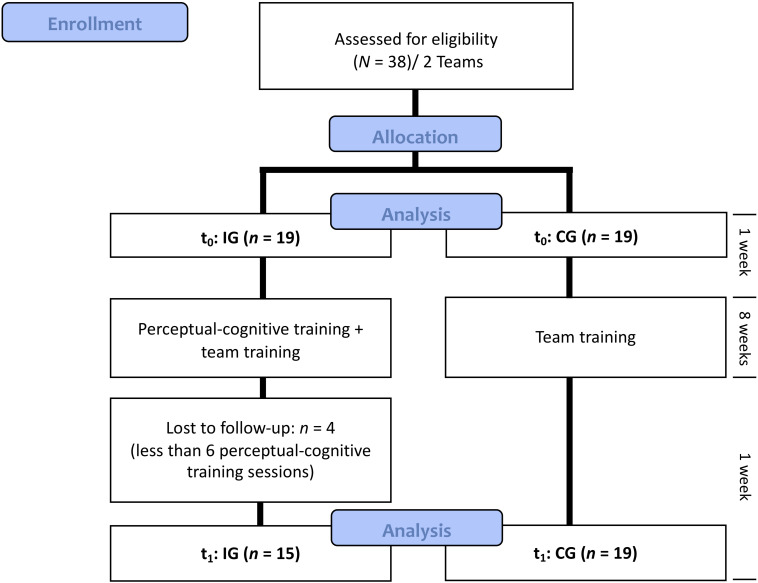
Study flowchart.

The study flowchart is shown in Figure 1. The experiment was a 2 (measuring point) × 2 (group) controlled design. Two elite U 14 teams were assessed for eligibility. Because of practicability, the teams and not the participants were randomized and allocated to the CG or the IG. In the week after pretest, the IG conducted the normal team training and the perceptual-cognitive training. The CG exclusively underwent usual team training. The perceptual-cognitive training was conducted once a week before the usual team training over 8 weeks. Because of participation in fewer than six perceptual-cognitive training sessions, four participants were lost to posttest. One week after the 8-week intervention, the posttest was completed by the IG and the CG.

### Measures

Peripheral vision was measured using the peripheral perception test (Schuhfried et al., 2011) of the Vienna Test System by Schuhfried GmbH. The test is computer-based and consists of a central tracking task and a peripheral perception task. The peripheral perception task is presented on two “peripheral displays” attached to the left and the right sides of the computer monitor. The peripheral displays consist of vertical and horizontal rows of light diodes (64 vertical diode columns and 8 horizontal diode rows). Stimuli are given by green lights traveling from the center of the visual field to periphery. Critical stimuli are shown as vertical blinking bars (blinking speed of 60 ms) on the peripheral display. Participants must react to critical stimuli by depressing a foot pedal. Time from appearance of the stimulus to pedal pressure is measured in seconds and defined as PRT. Simultaneously, a tracking task must be completed on the monitor (a red bullet must be followed with a crosshair in central FOV). Only those stimuli for which a reaction occurred and the crosshairs were within the tolerance range are used to calculate the reaction time. This requires the distribution of attentional resources between the tracking and the peripheral reaction task. Each time the pedal is pressed, the distance between the monitor and the subjects face is measured in centimeters by an ultrasound distance sensor. These measures are used for calculation of FOV. Peripheral reaction time is subdivided in peripheral reaction time right (RTR) and peripheral reaction time left (RTL). Peripheral reaction time is calculated by the median of PRTs for correct responses on right and left critical stimuli in seconds. Meaning that for RTL the stimuli appeared on the left side, whereas for RTR on the right side, respectively. The participants could react with their dominant foot (right or left foot). Field of vision is calculated by visual angle left (VAL) and visual angle right (VAR) and is given in degrees (n°). Tracking deviation (TD) is defined as deviation of crosshair from the tracking point in the tracking task and measured in pixel. Deviation at the moment of every critical stimulus (overall 40) is given as a mean. Primary outcomes were PRT, RTL, and RTR. Secondary outcomes were FOV, VAL, VAR, and the TD. Detailed description and reliability are given elsewhere (Schumacher et al., 2019).

### Perceptual-Cognitive Training

**FIGURE 2:**
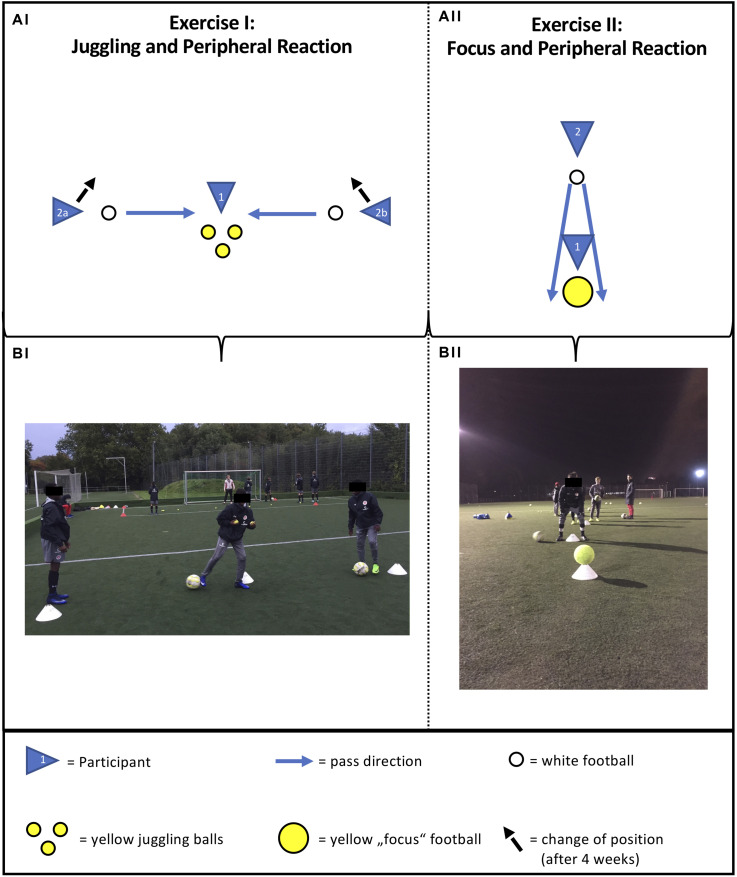
Schematic description (A_*I*_, A_*II*_) and photo (B_*I*_, B_*II*_) of exercise I and exercise II of perceptual-cognitive training.

One training session lasted about 20 min and always consisted of two exercises (Figure 2) and a parallel juggling training with yellow tennis balls.

In exercise I, called “Juggling and Peripheral Reaction,” player 1 tried to juggle while at the same time player 2a or player 2b passed a football to player 1. Because a three-ball juggling was not mastered at the beginning of the intervention, the participants started juggling with two balls. After 4 weeks, the three-ball juggling was introduced. While player 1 juggled, he had to play back the pass from player 2a or 2b directly and as fast as possible. Player 1 was instructed to do this using the outside of the foot. The distance between players 1 and 2a, respectively, 2b was 2.5 m. After 4 weeks, the position of players 2a and 2b was shifted approximately 5 degrees backward in order to include a progression in task difficulty. Every player did two sets of exercise I per week. In one set, every player was instructed to train on position 1 for 30 s. At the same time, the rest of the team carried out instructed juggling training (see background of B_*I*_ in Figure 2). The duration of the juggling training was 15 min per week.

In exercise II, called “Focus and Peripheral Reaction,” player 2 was positioned 5 m behind player 1. Player 1 was instructed to focus a yellow football lying 2 m ahead. Player 2 was instructed to roll another football past player 2 on the left or on the right. When player 1 detected the ball, he had to react by stopping the ball with his right or left foot. Every player had to detect five balls per week (five times position 1).

During the intervention phase, the CG completed the usual team training. *t*_0_ measurements were conducted 1 week before the training intervention started. *t*_1_ measurements took place 1 week past the 8-week training intervention.

### Statistical Analysis

SPSS version 24.0.0.2 was used for all statistical analyses (SPSS, 2016). Differences between groups of participants and dropouts for baseline characteristic were calculated using Mann–Whitney *U* test. Interaction effects between measuring points (*t*_0_ and *t*_1_) and groups (IG and CG) for all independent variables (PRT, RTL, RTR, VAL, VAR, TD) were assessed using two-factor repeated-measures ANOVA. Within-subject effects effects were controlled with the Greenhouse–Geisser method, so that a valid *F* ratio could be obtained and the type I error rate was reduced. The α level was set at 0.05. Effect size was calculated by η^2^. According to Cohen boundaries (Cohen, 1988), the effect size of the two-factor repeated-measures ANOVA was set to 0.01 (small effect), 0.06 (moderate effect), and 0.14 (large effect). To control for differences between left- and right-footed players in IG at *t*_0_ and *t*_1_, a Mann–Whitney *U* test was used.

## Results

Baseline characteristics of IG and CG are depicted in Table 1. Analyses of differences between groups at baseline demonstrate that participants of the IG were significantly taller (*p* = 0.02) and heavier (*p* = 0.00). Furthermore, participants of the IG showed faster PRTs when stimuli appeared on the left side (*p* = 0.04). No further differences between groups at baseline were found. Because of low participation rate, four players were excluded in the IG (inclusion requirement: participation in at least six training sessions). There were no differences between the included players and the four dropouts.

**TABLE 1:** Baseline characteristic of participants and dropouts, differences between groups using Mann–Whitney *U* test (*p*).

	Intervention group (*n* = 19) Mean ± SD	Control group (*n* = 19) Mean ± SD	*U* test (*p*)	Dropouts (*n* = 4) Mean ± SD	*U* test (*p*)
Age (years)	12.7 ± 0.5	12.7 ± 0.5	0.80	13 ± 0.0	0.42
Height (cm)	168.3 ± 8.8	158.9 ± 7.4	0.02*	164.0 ± 5.5	0.87
Weight (kg)	54.0 ± 9.1	46.1 ± 6.4	0.00**	51.6 ± 8.7	0.80
Training (h/wk)	7.1 ± 1.7	7.4 ± 1.5	0.37	6.5 ± 1.0	0.34
Competition (h/wk)	1.6 ± 0.4	1.7 ± 0.7	0.73	1.8 ± 0.9	0.57
Dominant leg right (n/%)	10/52.6	9/47.4		2/50	
Dominant leg left (n/%)	9/47.4	10/52.6		2/50	
RTR (s)	0.78 ± 0.07	0.80 ± 0.10	0.45	0.77 ± 0.02	0.70
RTL (s)	0.73 ± 0.09	0.80 ± 0.10	0.04*	0.70 ± 0.03	0.09
VAR (n°)	90.37 ± 3.54	87.12 ± 10.07	0.80	91.60 ± 1.03	0.58
VAL (n°)	86.88 ± 7.55	86.92 ± 9.49	0.69	86.97 ± 13.0	0.20

Values shown are the mean and standard deviation (SD). Results of Mann–Whitney U test are presented with p-values. RTR, peripheral reaction time right; RTL, peripheral reaction time left; VAR, visual angle right; VAL, visual angle left; n°, visual angle in degree. *p ≤ 0.05, **p ≤ 0.01. Significant p values shown in bold numbers.

**FIGURE 3:**
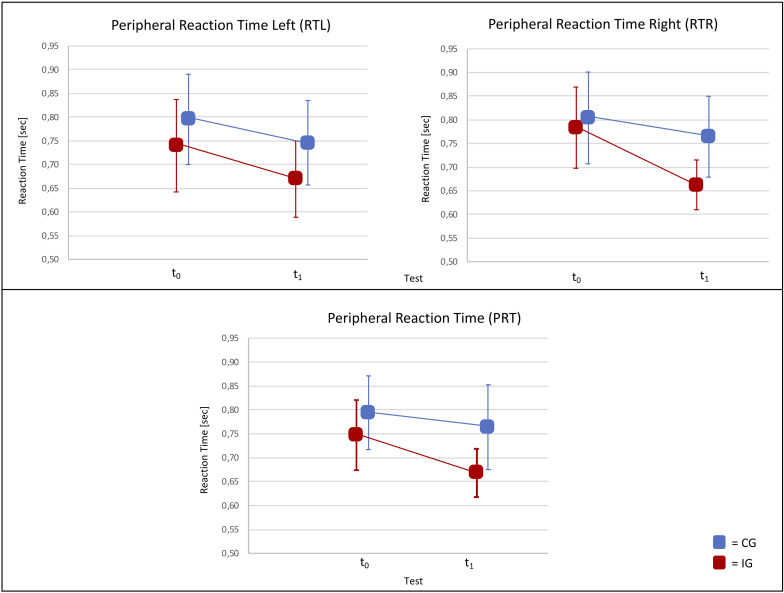
Reaction time (PRT, RTL, PRT) at pretest and posttest in the intervention and control groups. PRT, peripheral reaction time; RTR, peripheral reaction time right; RTL, peripheral reaction time left; *t*_0_, pretest; *t*_1_, posttest; IG, intervention group; CG, control group.

Results of analyses of interaction effects between measuring points (*t*_0_ and *t*_1_) and groups (IG and CG) for all independent variables (PRT, RTL, RTR, VAL, VAR, TD) are shown in Table 2 and Figure 3. Calculations with the two-factor repeated-measures ANOVA resulted in significant differences between groups and measuring points for PRT [*F*(1,32) = 4.85, *p* = 0.035] and RTR [*F*(1,32) = 9.63, *p* = 0.004], with the IG showing significant shorter reaction times. According to Cohen boundaries for PRT (η^2^ = 0.13) and RTR (η^2^ = 0.23), large effect sizes were observed. No significant interaction effect (time × group) was found for RTL [*F*(1,32) = 0.49, *p* = 0.49, η^2^ = 0.02]. Furthermore, no main effects were found for secondary outcome parameters. Analyses of differences between left- and right-footed players in the IG at *t*_0_ and *t*_1_ are shown in Table 3. Differences were found between left- (0.70 ± 0.06 s) and right-footed (0.63 ± 0.02) players at *t*_1_ for RTR (*U* = 48.00, *p* = 0.021). No significant differences were found between left- and right-footed player at *t*_0_ or *t*_1_ for other outcome parameters.

**TABLE 2:** Means and SD of outcome measures for groups at pretest and posttest and ANOVA (time × group).

Measure	Intervention group (IG) (*n* = 15)		Control group (CG) (*n* = 19)		ANOVA (time × group)
	Pretest (*t*_0_) Mean ± SD	Posttest (*t*_1_) Mean ± SD	Pretest (*t*_0_) Mean ± SD	Posttest (*t*_1_) Mean ± SD	
PRT (s)	0.75 ± 0.07	0.67 ± 0.05	0.79 ± 0.08	0.76 ± 0.09	*F*(1,32) = 4.97, *p* = 0.03, η^2^ = 0.14*
RTR (s)	0.78 ± 0.09	0.66 ± 0.05	0.80 ± 0.10	0.76 ± 0.09	*F*(1,32) = 9.56, *p* = 0.004, η^2^ = 0.23**
RTL (s)	0.74 ± 0.10	0.67 ± 0.08	0.80 ± 0.10	0.75 ± 0.09	*F*(1,32) = 0.48, *p* = 0.49, η^2^ = 0.02
FOV (n°)	176.90 ± 6.92	183.10 ± 3.96	174.03 ± 15.51	180.00 ± 12.04	*F*(1,32) = 0.01, *p* = 0.93, η^2^ = 0.00
VAR (n°)	90.04 ± 3.92	93.64 ± 3.61	87.12 ± 10.07	88.73 ± 9.84	*F*(1,32) = 3.57, *p* = 0.07, η^2^ = 0.10
VAL (n°)	86.85 ± 6.08	89.45 ± 1.76	86.92 ± 9.49	91.26 ± 4.15	*F*(1,32) = 0.51, *p* = 0.48, η^2^ = 0.02
TD (pixel)	12.07 ± 1.81	11.99 ± 2.17	12.88 ± 2.95	11.87 ± 3.44	*F*(1,32) = 0.89, *p* = 0.35, η^2^ = 0.03

Values shown are the mean and standard deviation (SD). Results of analysis of variance (ANOVA) (F) are presented with p-values and degrees of freedom. Effect size is shown by eta-square (η^2^). PRT, peripheral reaction time; RTR, peripheral reaction time right; RTL, peripheral reaction time left; FOV, field of vision; TD, tracking deviation; VAR, visual angle right; VAL, visual angle left; n°, visual angle in degree. *p ≤ 0.05, **p ≤ 0.01.

**TABLE 3:** Differences between left- and right-footed players for RTR and RTL in IG at *t*_0_ and *t*_1_ calculated with Mann–Whitney *U* test.

	Pretest (*t*_0_) Mean ± SD			Posttest (*t*_1_) Mean ± SD		
	Dominant leg left (*n* = 7)	Dominant leg right (*n* = 8)	*U* test (*p*)	Dominant leg left (*n* = 7)	Dominant leg right (*n* = 8)	*U* test (*p*)
PRT (s)	0.77 ± 0.09	0.73 ± 0.05	0.34	0.68 ± 0.06	0.66 ± 0.04	0.54
RTR (s)	0.83 ± 0.10	0.75 ± 0.05	0.09	0.70 ± 0.06	0.63 ± 0.02	0.02*
RTL (s)	0.75 ± 0.12	0.73 ± 0.11	1.00	0.68 ± 0.09	0.66 ± 0.08	0.40

Values shown are the mean and standard deviation (SD). Results of Mann–Whitney U test are presented with p-values. PRT, peripheral reaction time; RTR, peripheral reaction time right; RTL, peripheral reaction time left. *p ≤ 0.05. Significant p values shown in bold numbers.

## Discussion

The purpose of the present study was to investigate the effect of a soccer-specific perceptual-cognitive on-field training combining a sport-specific and a juggling task on the peripheral reaction abilities of highly talented male soccer players. The results of our study imply that perceptual-cognitive abilities can be improved by a sport-specific on-field training intervention. This intervention was associated with substantial decreases in PRT in the IG with no improvement for the CG. We observed significant differences between groups and measuring points for RTR and overall PRT. As can be seen in Table 2, the large effect in RTR accounts for the overall observed changes in PRT.

Our results are in line with previous investigations which have shown that perceptual-cognitive skills can be trained in sports (Savelsbergh et al., 2010; Schwab and Memmert, 2012; Romeas et al., 2016). Nevertheless, these studies analyzed computer-based and/or laboratory perceptual-cognitive training interventions. There is an ongoing discussion on different approaches in sports vision training such as naturalistic sports training to improve sport performance (Appelbaum and Erickson, 2016). Keeping in mind the call for ecological validity and transferability (Broadbent et al., 2015a; Hadlow et al., 2018; Renshaw et al., 2019), the conducted tasks of this study represent an on-field and sport-specific perceptual-cognitive training approach. Most notably, this is the first controlled trial to our knowledge investigating the effect of such an intervention on PRT. Moreover, a recent review amplifies that when investigating peripheral perception in sport, experimental control should not be disregarded (Vater et al., 2019b). In this study, we used a standardized testing procedure.

The intervention was composed of 5 min of exercise I, called “Juggling and Peripheral Reaction,” and exercise II, called “Focus and Peripheral Reaction.” While six players engaged in exercise I or II, the rest of the team performed the instructed juggling training (Figure 2). The duration of the juggling training was 15 min per week, but some participants unsystematically reported that they also trained at home. The difficulty of exercises I and II consisted of the random passing of the ball from the left or the right periphery while additionally juggling three tennis balls in exercise I. Tasks involving high contextual interference (random schedule) result in poorer performance during acquisition, but promote the long-term learning and transfer, when compared to low contextual interference (blocked schedule) (Lee, 2012). To what extent the contextual interference effect has an impact on perceptual-cognitive learning is not clarified yet (Memmert et al., 2009; Broadbent et al., 2015b). A major requirement of the intervention tasks conducted in this study was that participants had to distribute their attention to peripheral areas by focusing on a point between relevant information. This interaction between the location of the gaze and the distribution of attention might have had an impact on the effect of the intervention. To better understand the underlying mechanisms of peripheral vision in future studies, a dual-task approach should be pursued combining a monitoring with a detection task. This would allow to examine the processing of information via the fovea or peripheral vision and simultaneously investigate the location of gaze and the locus of attention (Vater et al., 2019b).

As anticipated, the effects of the intervention combining sport-specific and a juggling task in this study seem consistent with results of previous studies on the effects of juggling on perceptual-motor control (Beek et al., 2003). They discussed that juggling expertise was associated with an overall reduction in the degree to which the balls were visually tracked. They stated that probably with experience, jugglers relied more on peripheral vision than inexperienced jugglers. On the other hand, expert performers depend more on the sensations coming from the contact between the hands and the balls, and thus an expert immediately detects a slight deviation in the desired angle of release or in the energy imparted to the ball, whereas a novice has to see the effect of mistakes in the flight trajectories (Beek and Lewbel, 1995). Because the novice or intermediate jugglers in our study had to rely predominantly on their eyes, it is possible that the juggling training conducted in our study has led to an improvement in perception in the periphery, so that the perception and/or processing of stimuli and coupled motor response were improved. In order to verify such underlying mechanisms, studies on hemodynamic responses during juggling tasks should be conducted. Results of Carius et al. (2016) from investigations on the primary motor cortex might support the motor response hypothesis. They found that a higher level of expertise might be associated with lower hemodynamic responses during juggling tasks. These results could indicate that the juggling training performed may have had an impact on the primary motor cortex and thus led to improved reaction times. Nevertheless, underlying mechanisms in the visual cortex or the visuomotor network during perceptual-cognitive training remain to be explored in future studies. Moreover, to better understand processes during peripheral perception tests and during training, eye tracking methods should be considered as an additional measurement (Discombe and Cotterill, 2015; Vater et al., 2016; Kredel et al., 2017). However, based on our results, we cannot determine the effect of the juggling training alone on peripheral reaction. Thus, it cannot be ruled out that the effects in the PRT are due to exercise I, exercise II, the juggling task, or the combination. Therefore, future studies should conduct controlled experiments with exercise I, exercise II, and a juggling task as single interventions. Nevertheless, in light of the relatively low dose of exercises I and II, one could amplify that the described effect might be mainly due to the juggling training.

Considering that we found no significant interaction effect (time × group) for RTL, our findings suggest that the perceptual-cognitive training conducted had an influence only on the right-sided peripheral reaction of young, highly talented soccer players. The significant interaction effects found for RTR in contrast to RTL raise the question whether the results are associated with differences in left- and right-footedness. Analyses of the baseline characteristics showed no differences in the footedness between the IG and the CG at *t*_0_. Surprisingly, we found differences between left- and right-footed players in the IG only postintervention for RTR. This illustrates that postintervention right-footed players in the IG showed significantly shorter reaction times than left-footed players when the stimulus appeared from the right side. Prior to the intervention, no significant differences in PRT between left- and right-footed players could be found for either left-sided stimuli or right-sided stimuli. These findings suggest that the improvement in right peripheral reaction in the IG might be due to changes in the reaction time of right-footed players in the IG. This is remarkable in regard to studies assuming superior motor abilities in left-footed as well as left-handed athletes (Dane and Erzurumluoglu, 2003; Hagemann, 2009; Loffing et al., 2010, 2012; Breznik, 2013; Tran and Voracek, 2016). Nevertheless, the effect of the training intervention carried out seems to have had a greater impact on right-footed players. This phenomenon should be investigated in further studies. In this context, investigations on hemodynamic responses of the cortex might again be of special interest.

Improvements in PRT after the on-field intervention was conducted are important against the background of earlier findings (Zwierko, 2007). Using the computer-based Vienna Test System, Zwierko (2007) showed that Handball players had significantly shorter response time to stimuli appearing in the peripheral FOV compared to non-athletes. These results indicate that peripheral reaction abilities measured with the Vienna Test System might be associated with expertise in sports. The herewith conducted perceptual-cognitive on-field training led to improvements in peripheral reaction abilities. To what extent this leads to soccer-specific improvements remains to be investigated.

These findings could suggest a connection between general perceptual-cognitive abilities and sport-specific skills. In this context, it also seems possible to discuss the expert-performance approach in the context of the cognitive component skill approach. As introduced above, the cognitive component skill approach examines the relationship between sports expertise and performance of non–sport-specific, general cognition (Nougier et al., 1991). Studies based on the cognitive component skill approach provide heterogeneous results (Mcauliffe, 2004; Baláková et al., 2015), and thus the role of the cognitive component skill approach in sports expertise research is not clarified yet. The expert-performance approach analyzes the experts under a sport-specific, ecologically valid context (Gilbert et al., 2016). The analyses of sport-specific tasks should help to identify the underlying mechanisms of sport expertise and to identify and develop training forms and activities leading to the adoption and development of these mechanisms (Ericsson and Smith, 1991). Both approaches seem to be highly relevant for the clarification of the highly complex sporting expertise. Therefore, future investigations should examine the extent to which general perceptual-cognitive abilities may underlie sports expertise and in which context the specificity of the sport is decisive in order to differentiate expertise performance in terms of perceptual-cognitive abilities.

Our results provide first evidence that an on-field training including a juggling task can improve PRT in elite soccer players. However, some limitations are worth noting. An aspect to consider is that the participants in the IG were significantly taller and heavier than those in the CG at baseline. Physical development could have an influence on the effect of a perceptual-cognitive intervention in addition to calendar age. Furthermore, because of practical considerations, randomization was limited to existing teams and could thus have led to further selection bias. In the IG, four elite soccer players were lost to *t*_1_ measurements as they participated in fewer than six training sessions. Nevertheless, these dropouts did not significantly differ from the included participants. A confounding variable might be the content of regular training. The regular team training of the intervention and CG was not recorded. It is possible that the IG carried out exercises in the regular training that led to an improvement of the peripheral reaction. Nevertheless, the coaches of both teams reported the same number of training sessions and similar training content. Moreover, some participants unsystematically reported that they also trained juggling at home. This might have biased our results. In order to generalize present results, it will be important to increase the statistical power with a larger sample in future studies. Although this study hints at the existence of effective perceptual-cognitive on-field training for general PRT, further confirmatory work is needed. Future studies should also investigate activity changes in the motor cortex under perceptual-cognitive training conditions before, during, and after the intervention period and control for eye movements during peripheral perception training and testing. Moreover, age (biological and calendric age) and positional influences should be considered (Schumacher et al., 2018).

These examinations deliver noteworthy practical applications: (1) The conducted sport-specific perceptual-cognitive training is feasible without substantial additional resources within usual soccer training. (2) A dose of 20 min per week (over 8 weeks) of perceptual-cognitive training led to significant changes in perceptual-cognitive abilities in a sample of U 14 players.

## Conclusion

In conclusion, the present study delivers several findings on the effect of a soccer-specific perceptual-cognitive on-field training combining a sport-specific and a juggling task on the peripheral reaction abilities of elite U 14 male soccer players. A significant interaction effect between groups and measurement points for RTR and overall PRT with substantial decreases in PRT in the IG and no improvement for the CG was detected. We also found differences between left- and right-footed players in the IG only postintervention for RTR. Aforementioned results indicate that improvement in right peripheral reaction might be due to changes in the reaction time of right-footed players in the IG. These results provide first evidence that a perceptual-cognitive on-field training including a juggling task can improve PRT in elite U 14 soccer players. Nevertheless, further studies are needed to clarify the effect of sport-specific on-field training interventions on PRT. These analyses should consider the influence of lateralization on effectivity of perceptual-cognitive on-field training.

## Data Availability Statement

The raw data supporting the conclusions of this article will be made available by the authors, without undue reservation.

## Ethics Statement

The studies involving human participants were reviewed and approved by the local ethic committee of the Faculty of Psychology and Human Movement Science, University of Hamburg. Written informed consent to participate in this study was provided by the participants’ legal guardian/next of kin.

## Author Contributions

NS and K-MB: conceptualization. NS: data curation, formal analysis, investigation, methodology, project administration, and writing—original draft. RR: resources. RR and K-MB: supervision. All authors contributed to the article and approved the submitted version.

## Conflict of Interest

The authors declare that the research was conducted in the absence of any commercial or financial relationships that could be construed as a potential conflict of interest.
